# A Rare Case of Primary Small Cell Carcinoma of Esophagus

**DOI:** 10.7759/cureus.17190

**Published:** 2021-08-15

**Authors:** Snehasis Das, Naveen Kumar Gaur, Oseen Shaikh, Gopal Balasubramanian, Sreerekha Jinkala

**Affiliations:** 1 Surgery, Jawaharlal Institute of Postgraduate Medical Education and Research, Puducherry, IND; 2 Pathology, Jawaharlal Institute of Postgraduate Medical Education and Research, Puducherry, IND

**Keywords:** small cell carcinoma, neuroendocrine tumor, carcinoma esophagus, dysphagia, synaptophysin, chromogranin

## Abstract

Primary small cell carcinoma of the esophagus is a rare, highly aggressive disease with a poor prognosis. A definitive diagnosis is made by histopathological study. As the disease is usually metastatic, palliative chemoradiotherapy is the usual treatment. We present a case of a 57-year-old female presenting with dysphagia. The patient underwent imaging studies showing the growth at the gastro-esophageal junction, with extensive abdominal lymph node metastasis and liver and lung metastasis. Biopsy was suggestive of small cell carcinoma of the esophagus. The patient underwent a feeding jejunostomy and was planned for chemoradiotherapy. Primary small cell carcinoma of the esophagus is an infrequent entity. As the disease is usually diagnosed at a later stage, the prognosis is inferior and abysmal.

## Introduction

Small cell carcinoma (SCC) is a rare primary esophageal tumor, accounting for less than 1% of all esophageal malignancies [1]. These tumors are high-grade tumors with a poor prognosis. Patients usually present with dysphasia with early metastasis to distant organs. Endoscopy and imaging studies help in diagnosis. However, histopathological examination is the only definitive way of diagnosis. The immunohistochemical examination is beneficial in diagnosing the SCC of the esophagus. Most cases of SCC of the esophagus are metastatic at the time of diagnosis and hence have limited treatment options and a fatal prognosis [1]. Surgical treatment is rarely helpful as patients usually have metastatic disease. Patients are usually treated with chemoradiotherapy. We present a 57-year-old female, diagnosed with metastatic SCC of the esophagus, managed with palliative chemoradiotherapy and feeding jejunostomy.

## Case presentation

A 57-year-old female with no co-morbidities presented with complaints of upper abdominal pain with progressive dysphagia for one month. The pain was mainly in the epigastrium, mild to moderate in severity, relieving analgesic medications. The patient had progressive dysphagia for the last three months and was losing appetite with significant weight loss over the last one month. On examination, the patient had pallor, was mildly dehydrated, and vitals were stable. There was mild tenderness in the epigastrium, with multiple round nodular hard masses in the epigastrium and umbilical region.

**Figure 1:**
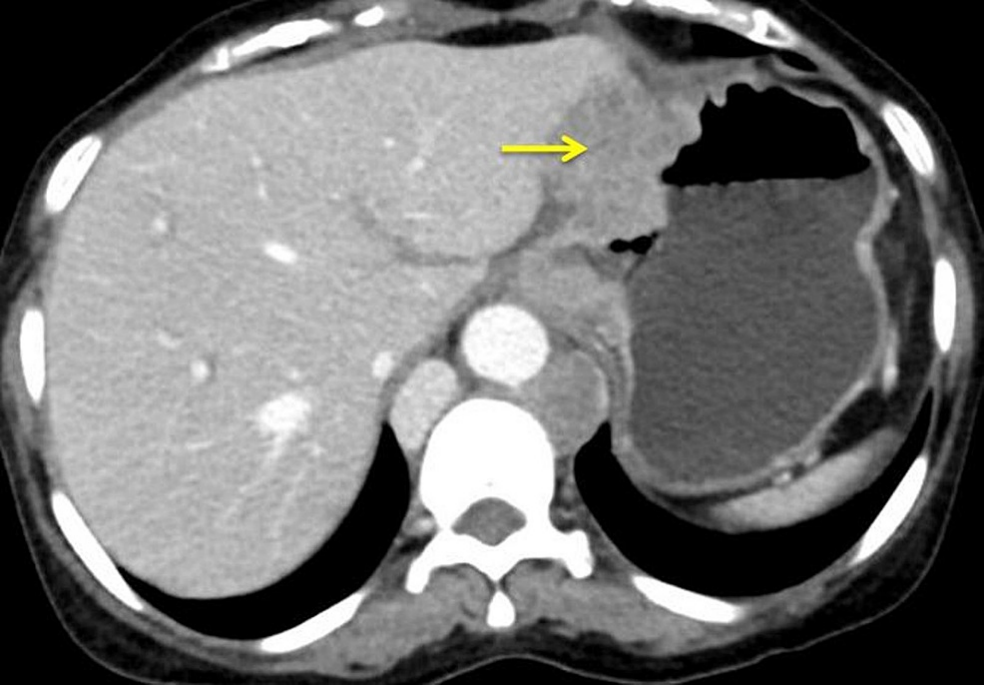
Contrast-enhanced computed tomography (axial view) showing growth extending to the lesser curvature (arrow).

The patient had mild anemia (8.9 g/dl) and leukocytosis. The liver function test (LFT) and renal function test (RFT) were normal. The abdomen's ultrasound showed multiple para-aortic nodes, with the largest measuring almost about 1.3 cm, without any evidence of free fluid in the abdomen and metastasis to the liver. Contrast-enhanced computed tomography (CECT) of abdomen and thorax showed circumferential, asymmetric, irregular, heterogeneously enhancing nodular wall thickening, involving the mid, distal esophagus, gastroesophageal junction, cardia, and the body of the stomach predominantly along the lesser curvature (Figure 1).

**Figure 2:**
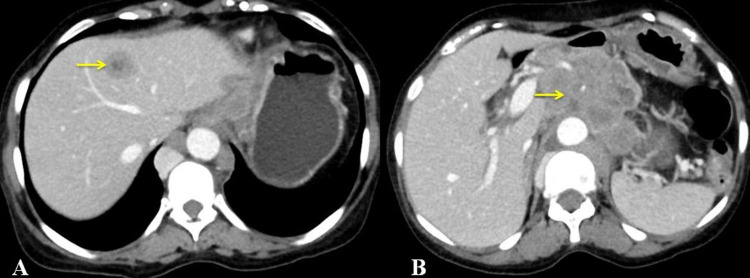
Contrast-enhanced computed tomography (axial view) showing (A) metastatic lesion in the liver suggested by target lesion (arrow) and (B) confluent large heterogeneously enhancing lymph nodal mass (arrow).

Multiple enlarged, heterogeneously enhancing, peri-esophageal, perigastric nodes were present. Large confluent heterogeneously enhancing lymph nodal mass predominantly comprises periportal nodes, peripancreatic nodes, perigastric nodes, celiac axis nodes, superior mesenteric artery nodes, retrocrural nodes, and paraaortic nodes, together with measuring 8.1 cm x 7.8 cm x 13.2 cm. Heterogeneously enhancing lesion (target or bulls' eye) was noted in the liver and multiple tiny parenchymal and subpleural nodules were noted in the bilateral lung parenchyma, suggestive of metastasis (Figure 2).

Upper gastrointestinal endoscopy showed an ulceroproliferative lesion present in the distal esophagus, causing near-total luminal stenosis. The scope could not be passed into the stomach. Multiple biopsies were taken from the lesion and sent for histopathological examination.

**Figure 3:**
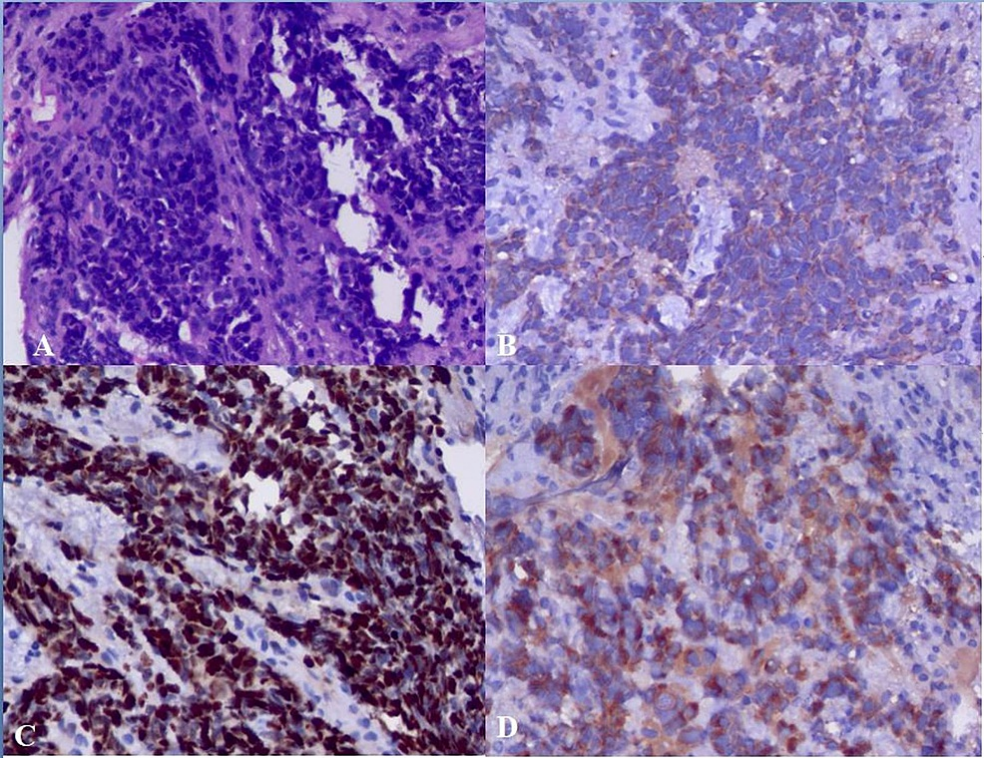
Cytology and immunohistochemistry images showing (A) tumor cells arranged in the nest having scant to absent cytoplasm and hyperchromatic nuclei, (B) cluster of differentiation 56 (CD56) immunostaining positivity, (C) Ki67 more than 95%, and (D) synaptophysin staining positivity.

Histopathologic examination showed tumor cells arranged in the nest and has scant to absent cytoplasm and hyperchromatic nuclei. Immunohistochemical examination showed that tumor cells were positive for synaptophysin and cluster of differentiation 56 (CD56) but negative for p63. Chromogranin staining was noncontributory. Ki67 index was 95% (Figure 3).

**Figure 4:**
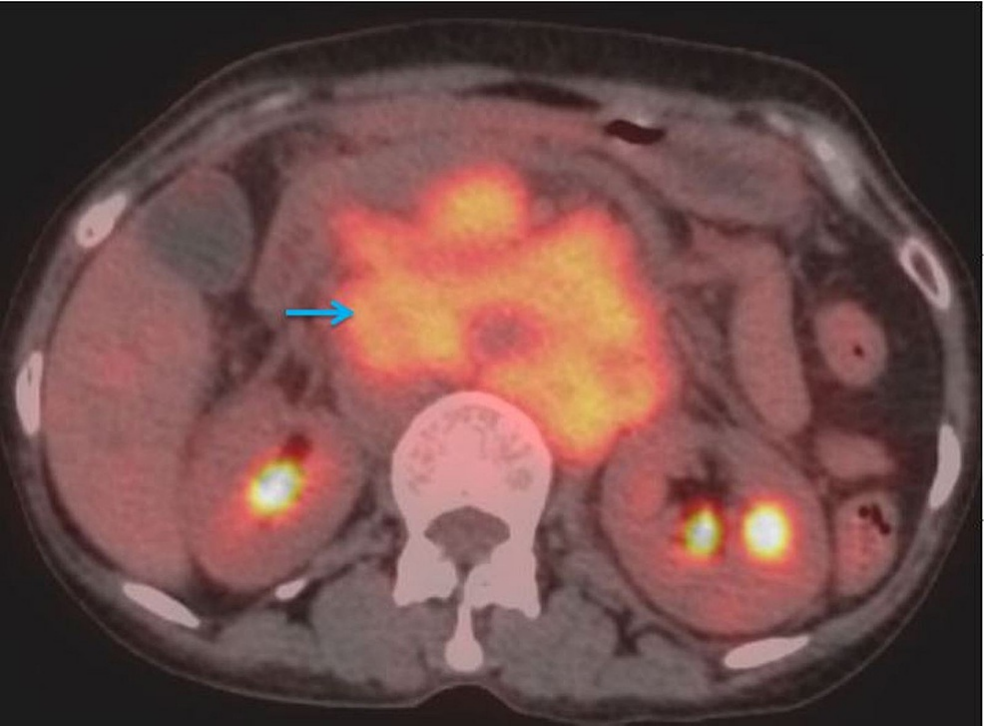
Positron emission tomography-computed tomography (axial view) showing metabolically active, large, conglomerated multiple lymph nodal mass (arrow).

Positron emission tomography-computed tomography (PET-CT) showed metabolically active primary disease involving cardia and the body of the stomach, extending till the gastroesophageal junction infiltrating the distal esophagus. Metabolically active multiple perigastric nodes, retroperitoneal lymph nodes, para-esophageal nodes, left upper paratracheal nodes, and right segmental lymph nodes were present. Solitary liver metastases and multiple bilateral lung metastases were present (Figure 4).

The patient was diagnosed with a case of metastatic SCC of the esophagus. We discussed the case on the tumor board, and the patient was planned for palliative chemotherapy (cisplatin and etoposide) considering the metastasis disease and poor prognosis. However, as the patient had absolute dysphagia, a feeding jejunostomy was done. The patient was started on palliative chemotherapy for small cell carcinoma of the esophagus. The patient was followed-up for the next three months and did not have any complications.

## Discussion

SCC of the esophagus, a rare neuroendocrine neoplasm (NEN), is usually seen in the age group of 29 years to 88 years of age [1]. SCC of the esophagus is more common in males [2]. The most common site of the SCC is the lungs, followed by the gastrointestinal tract, salivary glands, pharynx, larynx, and cervix [3]. The SCC of the esophagus is very rare, and establishing the incidence of the disease is difficult. However, in one study, it is found that the SCC constitutes less than 1% of all the esophageal malignancies [4].

Few studies have shown an association of neuroendocrine tumors with Von Hippel-Lindau (VHL) disease and multiple endocrine neoplasias 1 (MEN1). However, there is no direct association described in the literature with the SCC of the esophagus. Few have proposed that smoking and old age are risk factors for SCC [5].

The cellular origin of the esophageal SCC is controversial and is theorized to arise from the argyrophilic cells of Kulchitsky present in the mucosa. Because small cell carcinoma of the esophagus behaves almost similar to the small cell carcinoma of the lung, the diagnostic and treatment modalities are also tethered accordingly. NEN of the gastroenteropancreatic (GEP) was staged in 2006 and 2007 by the European Neuroendocrine Tumour Society (ENETS) based on the tumor's histological grading, including mitotic count, and the Ki-67 labeling index [5]. They classified the tumors as well-differentiated and poorly differentiated tumors. The poorly differentiated neoplasm, also called neuroendocrine carcinoma, has been further sub-classified as large cell carcinoma (>20 mitoses/mm^2^ and >20 Ki67 indexes) and small cell carcinoma (>20 mitoses/mm^2^ and >20 Ki67 indexes), both of which are considered as high grade.

Patients with SCC of the esophagus present with vague, varied symptomatology, including progressive dysphagia, weight loss, loss of appetite, and dyspepsia. These tumors involve the distal esophagus with or without extension into the gastrointestinal junction, which causes them to become symptomatic early. The mean interval from the onset of symptoms to a diagnosis has been reported to be around four months [1]. When diagnosing a small cell carcinoma with its primary at the esophagus, metastatic dissemination is the usual trend for around 31% to 90% of the cases. It thereby incurs a fatal prognosis from the start. The liver, adrenals, and lymph nodes are the most common sites [1]. In our case, the patient had metastasis to the abdominal lymph nodes, liver, and lungs.

Patients usually undergo upper gastrointestinal endoscopy (UGIE) because of dysphagia. UGIE shows the presence of the growth, its location, amount of narrowing of the esophagus, and most importantly, a biopsy can be taken from the tumor. In our patient, there was ulceroproliferative growth in the distal esophagus, causing a partial luminal obstruction.

CECT thorax, abdomen, and pelvis show the site of the growth and stage of the disease and detect the metastasis site in other organs. In addition, PET-CT is used to detect distant metastasis. However, the imaging methods cannot differentiate SCC from the other primary esophageal malignancy. However, the presence of extensive metastasis may be suggestive of the SCC of the esophagus. In our case, the patient had growth in the gastroesophageal and extensive metastasis to the abdominal lymph nodes. There was also evidence of liver and lung metastasis.

Histopathological examination is the only definitive way to diagnose the SCC of the esophagus. SCC of the esophagus is a high-grade neoplasm with >20 mitoses/mm^2^ and >20 Ki67 indexes. Cells may be arranged in a trabecular or ribbon pattern, an acinar or gland-like pattern, and a diffuse or solid pattern. The cells are monotonous with moderate granular cytoplasm, round eccentric nuclei with salt and pepper chromatin. Cells are small to medium-sized, round to oval cells with scant cytoplasm, and hyperchromatic nuclei with indistinct nucleoli. Immunohistochemical staining with synaptophysin and chromogranin A (CgA) are usually positive, although CgA may sometimes be focally positive or absent. Synaptophysin being a more sensitive stain, while chromogranin A is more specific. Other tumor markers like neuron-specific enolase (NSE), protein gene product 9.5, CD56, keratin, epithelial membrane antigen, and thyroid transcription factor 1 may be expressed. However, their specificity and clinical importance are less well established [6].

As the small cell carcinoma of the lung and the esophagus share the same genetic lines, a multimodality approach involving chemotherapy with cisplatin and etoposide with radiotherapy has been seen to offer the best chances of long-term survival [7]. Due to the absence of long-term follow-up study in such patients due to apparent fatality, there are no proper guidelines for endoscopic surveillance or radiological follow-up in patients who have survived beyond the expected duration of survival post-diagnosis. Median survival of less than one year, approximately eight months with limited disease, and three months with the extensive disease have been reported [8,9]. Even after surgery in limited disease, the recurrence rates are seen to be overwhelming [10]. In our case, the patient had received palliative chemoradiotherapy and feeding jejunostomy with the best supportive care. However, the patient was advised to follow-up in the palliative clinic after the surgery.

## Conclusions

Small cell carcinoma is the rarest and the most fatal in prognosis among all other subtypes of esophageal malignancies. The presence of extensive metastasis in the initial scans should raise suspicion of the SCC of the esophagus. Being usually diagnosed at an advanced stage, a multimodal chemoradiotherapy approach is seen to give the best response. Surgical options are only viable for the limited disease, but the recurrences post-surgical excision is high. In the premise of all the possible treatment options, the diagnosis remains belligerently fatal with a mean survival of less than a year.
